# Wearable IMU-Derived Kinematic Reference Profiles of Lower-Limb Kick and Wipe Gestures for Contactless Automotive Tailgate Activation

**DOI:** 10.3390/s26144469

**Published:** 2026-07-14

**Authors:** János Dreveton, Moritz Labetzsch, Tim Gocke, Torsten Bertram

**Affiliations:** 1 Institute of Control Theory and Systems Engineering (RST), TU Dortmund University, Otto-Hahn-Str. 8, 44227 Dortmund, Germany; torsten.bertram@tu-dortmund.de; 2 BMW Group, Petuelring 130, 80809 Munich, Germany; moritz.labetzsch@bmw.de (M.L.); tim.gocke@bmw.de (T.G.)

**Keywords:** wearable inertial sensors, inertial measurement unit, lower-limb kinematics, human movement analysis, kick gesture, wipe gesture, contactless tailgate activation, automotive human–machine interaction, gesture recognition, sensor-system evaluation

## Abstract

Contactless automotive tailgate activation relies on recognizing intentional lower-limb gestures near the rear bumper, yet these systems are developed and evaluated from sensor-specific recordings rather than from the underlying human movement, so quantitative, sensor-independent kinematic reference profiles for these gestures are lacking. This study establishes wearable inertial measurement unit (IMU)-derived reference profiles of two tailgate-activation gestures: a forward kick and a lateral wipe. Lower-body motion was recorded in 56 adult participants using a seven-sensor Xsens Awinda configuration under application-oriented conditions, yielding 6879 segmented movements. To the best of our knowledge, this is among the most extensive of such datasets, providing a sensor-independent, joint- and segment-level movement reference. Both gestures shared a common sagittal structure dominated by knee, ankle, and hip flexion/extension, with mean knee flexion/extension of $45.9^{\circ }$ for kick and $42.3^{\circ }$ for wipe movements. Wipe gestures differed through markedly larger non-sagittal components, with hip abduction/adduction of $17.2^{\circ }$ versus $7.7^{\circ }$ and ankle internal/external rotation of $16.6^{\circ }$ versus $8.7^{\circ }$, confirmed in every participant (p<0.001). Foot-segment kinematics showed the highest velocities, with a mean resultant foot velocity of approximately 1.8m/s. These profiles provide a quantitative biomechanical basis for benchmarking gesture-recognition sensor systems, informing detection-window and threshold selection, and enabling standardized, repeatable testing of contactless automotive HMI systems.

## 1. Introduction

Wearable inertial measurement units (IMUs) have become established tools for quantitative human movement analysis because they enable mobile, non-invasive and scalable acquisition of body-segment kinematics beyond conventional laboratory-based optical motion-capture systems [1,2]. By combining accelerometer, gyroscope and, in many systems, magnetometer data, wearable inertial sensor systems can estimate segment orientations, joint angles, and movement dynamics during natural movement execution [1,3]. Their independence from external camera infrastructure makes them particularly suitable for applications in which optical motion capture is limited by marker occlusion, restricted installation space, setup complexity, or the need to record movements in realistic environments [4,5]. Accordingly, wearable IMUs have been widely adopted in gait analysis, rehabilitation monitoring, ergonomics, sports biomechanics, and human activity recognition [2,6].

Most established IMU-based movement analysis applications focus on cyclic locomotion, clinical motor assessment, rehabilitation exercises, or performance-oriented sport movements [2,4,7]. These movements are typically repetitive, task-standardized, or embedded in well-defined biomechanical frameworks, facilitating the segmentation, comparison, and interpretation of sensor-derived kinematic variables. In contrast, discrete, intentional, and non-periodic lower-limb gestures remain comparatively under-characterized, although gesture-based interaction is increasingly used as an intuitive input modality for human–machine interaction (HMI) [8,9]. Unlike gait cycles or athletic kicking techniques, lower-limb HMI gestures are usually short, submaximal, and contact-free. They are performed without an external target, impact event, or strictly prescribed movement pattern, and their execution may depend on user strategy, stance position, interaction geometry, and everyday constraints. Dedicated kinematic reference profiles are therefore required to describe these movements quantitatively and to make them usable for sensor-system development, validation, and repeatable functional testing.

A practically relevant example of lower-limb gesture-based HMI is contactless automotive tailgate activation. Such systems allow users to open or close a vehicle tailgate by performing a foot gesture near the rear bumper, typically when their hands are occupied by luggage, groceries, or other objects. Current systems use vehicle-integrated sensing modalities such as capacitive or radar-based sensors to detect valid lower-limb gestures and reject unintended movements [10,11,12]. Radar-based approaches are particularly attractive because they can be integrated compactly behind vehicle body structures and provide motion-related observables such as range and Doppler information [10,11]. Existing studies have demonstrated that foot gestures can be recognized using Doppler signatures, hidden Markov models, deep learning architectures, or other machine-learning-based approaches [10,11,12].

However, the current literature on contactless tailgate activation and foot-gesture recognition primarily addresses the technical classification problem. Recognition algorithms are typically developed and evaluated based on vehicle-integrated sensor signals, whereas the underlying human movement itself is only implicitly defined by the recorded examples. As a result, detection thresholds, feature windows, training datasets, and functional test procedures are often based on empirical gesture executions rather than on quantitative human kinematic reference data. This creates a gap between sensor-signal classification and human movement analysis, since sensor systems are optimized to recognize a target motion that has not yet been systematically described at the joint and segment levels. Wearable IMU-derived reference profiles can help close this gap by providing quantitative descriptions of gesture timing, joint range of motion, angular velocities, segment velocities, and inter-individual variability. This gap is practically relevant because empirical or proprietary recordings describe one specific sensor in one mounting position, so the derived thresholds do not transfer to other modalities, positions, or vehicle classes, and there is no sensor-independent reference for comparing modalities or for defining target trajectories for repeatable functional testing, including robotic reproduction of the gesture.

Biomechanical data from sport-specific kicking movements cannot be directly transferred to this application. Soccer instep kicks are performance-oriented movements designed to maximize ball velocity and are characterized by a proximal-to-distal acceleration sequence, high foot velocities, and a decisive foot–ball impact event [13]. Combat-sport kicks are similarly optimized for rapid execution, target contact, and high impact force [14,15]. In contrast, tailgate activation gestures are everyday interaction movements performed at submaximal intensity, close to a vehicle, and without physical contact. Their purpose is not to generate maximal end-effector velocity or impact force but to produce an intentional and recognizable motion within a constrained interaction area. Moreover, wipe-like gestures have no direct equivalent in conventional gait or sport-kicking models because they combine lateral foot motion with an HMI-specific activation intent rather than locomotor progression or striking performance.

Therefore, the aim of this study was to establish wearable IMU-derived kinematic reference profiles of lower-limb kick and wipe gestures used for contactless automotive tailgate activation. Lower-body motion was recorded in a heterogeneous adult participant group using a seven-sensor IMU configuration while participants performed both gesture types under application-oriented conditions, including different starting positions, trajectory variants, and loading states. The analysis focused on temporal characteristics, joint-level range of motion (RoM), angular velocities and accelerations, segment velocities and accelerations, position-dependent variability, demographic variability, and gesture-specific principal movement patterns. The main contributions of this work are the large-scale IMU-based characterization of lower-limb HMI gestures and the derivation of quantitative kinematic reference profiles for kick and wipe movements at the joint and segment levels. To the best of our knowledge, this dataset, with 56 analyzed participants and 6879 segmented movements, is among the most extensive reported for contactless tailgate-activation gestures, and in contrast to the sensor-specific datasets used in previous work in this application area, it provides a sensor-independent kinematic reference at the joint and segment levels. In addition, this study assesses gesture variability relevant for the design, benchmarking, and repeatable functional testing of contactless tailgate activation systems. The resulting profiles should be interpreted as a biomechanical reference layer rather than as sensor-specific detection thresholds, because the mapping between IMU-derived kinematics and vehicle-integrated sensor responses depends on the sensing modality, sensor placement, and vehicle geometry.

## 2. Materials and Methods

### 2.1. Study Design and Participants

This study was designed as an experimental human movement analysis study to characterize the kinematic execution of lower-limb gestures used for contactless automotive tailgate activation. Lower-body motion was recorded using a wearable inertial motion-capture system while participants performed two discrete foot gestures—a kick gesture and a wipe gesture—under controlled but application-oriented interaction conditions. The analysis focused on temporal characteristics, joint and segment kinematics, inter-individual variability, and gesture-specific principal movement patterns.

The study protocol was designed to capture natural gesture executions rather than to enforce a strictly prescribed movement pattern. Participants were therefore instructed to perform the gestures in a way that reflected intuitive use of a hands-free tailgate activation system. To capture a broad range of execution strategies, participants with and without prior familiarity with contactless tailgate activation were included.

The target number of participants was derived from a standard sample-size estimation for a proportion in a large population [16]:

(1) $$n=\frac{z^{2}\;p\;\left(1-p\right)}{e^{2}},$$

where *p* denotes the assumed proportion, *z* the z-value corresponding to the confidence level, and *e* the margin of error. Assuming a proportion of p=0.5, a 90% confidence level with a corresponding z-value of z=1.65 and a margin of error of e=0.1 yields about 69 participants. This estimation was used as a pragmatic criterion to determine an adequate participant number for the descriptive, reference-generating aim of this study, rather than to estimate a specific population proportion, and the 90% confidence level reflects the practical constraints of the application-oriented measurement setup. Of the 69 recorded participants, 13 were excluded because no valid recording remained, mostly due to sensor-related problems such as magnetic disturbance or sensor detachment, or because parts of the protocol could not be carried out. Exclusion was always applied to a complete recording rather than to individual movements, and all movements within a valid recording were retained. Within the analyzed participants, a further 26 individual recordings were excluded for the same reasons. The final analyzed sample comprised n=56 adult participants, including $n_{m}=42$ male and $n_{f}=14$ female participants, with a mean age of 46.7±14.0 years and an age range of 21–78 years. The analyzed sample therefore fell below the target of approximately 69 participants indicated by Equation (1), an aspect that is addressed in the limitations. Participants were recruited to cover a broad range of age groups and anthropometric characteristics relevant to the automotive segment. In this context, the term heterogeneous refers to the diversity of the participant sample with respect to age (ranging from 21 to 78 years), male and female sex, a wide range of lower-body anthropometric measurements (as summarized in Table 1), and differing prior familiarity with contactless tailgate activation, since both experienced and inexperienced users were included. The age and sex distribution is summarized in Table 2.

Anthropometric measurements of the lower body were recorded before data collection to document participant variability relevant to lower-limb gesture execution. Measurements were obtained with participants wearing clothing and shoes, reflecting the practical use case of contactless tailgate activation. The descriptive anthropometric data are reported separately for male and female participants in Table 1.

All participants provided written informed consent before data collection. The study protocol, data protection procedures, and participant information were reviewed and approved by the BMW Group ethics committee, data protection office, and works council. All data were anonymized before processing and analysis.

### 2.2. Experimental Setup and Gesture Tasks

**Figure 1:**
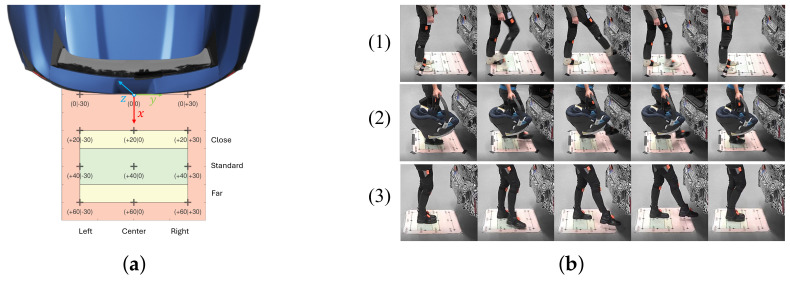
Experimental setup for gesture data collection. (**a**) Positioning template behind the rear bumper. (**b**) Representative gesture executions, including unloaded kick (**1**), loaded kick (**2**), and unloaded wipe (**3**).

Data collection was performed in an application-oriented experimental setup using a test vehicle (BMW X2, U10) and a predefined positioning template placed behind the rear bumper. The template defined the participant’s starting position relative to the vehicle by combining three lateral offsets—namely, left, center and right—with three distances from the rear bumper. The lateral offsets were ±30 cm relative to the vehicle centerline, and the distances from the rear bumper were 20, 40, and 60 cm. These positions, including the lateral offsets and bumper distances defined by the positioning template, followed the specification of the vehicle-integrated sensor supplier and were selected to cover the relevant interaction area behind the vehicle and to capture variability caused by different user positions relative to the sensing area. A schematic overview of the positioning template and representative gesture executions is shown in Figure 1.

Each participant performed two gesture types: a kick gesture and a wipe gesture. Participants were instructed to execute the gestures in a natural and intuitive manner, as they would use them to activate a hands-free tailgate system. No sport-like movement pattern, target impact, or prescribed end-effector velocity was imposed.

Before the recording sequence, each participant performed one self-selected execution of each gesture type without detailed technical instruction. This initial execution served as a familiarization trial and as a reference for the participant’s uninstructed movement strategy before the study protocol influenced motor planning.

During the standardized recording sequence, all predefined positions were recorded for both gesture types. Movements were performed with the left and right leg in alternating order, with four repetitions per leg and position, chosen as a compromise between capturing within-participant variability and keeping the overall protocol duration manageable across the large number of position, gesture, and loading combinations. Both legs were recorded, and the analysis was performed on the executing leg of each gesture, because the contactless tailgate activation is triggered by the gesture-performing leg while the contralateral stance leg does not contribute to the activation movement. Leg dominance was not separately recorded, as each participant performed the gestures with both the left and the right leg and both were analyzed as executing legs. The order of recording conditions was randomized for each participant to reduce learning and sequence effects.

The central standard position at a distance of 40 cm and zero lateral offset (+40|0) was additionally recorded in three trajectory variants. These variants comprised an uninstructed natural execution, a high variant in which the foot was moved close to the lower edge of the rear bumper, and a low variant in which the foot trajectory was performed close to the ground. These variants were included to capture a broader range of practically relevant gesture amplitudes and foot trajectories. The left and right standard positions were used to capture diagonal movement geometries relative to the vehicle centerline.

For wipe gestures, clockwise and counterclockwise movement directions occurred, depending on the participants’ intuitive execution strategy. These directions were treated separately in time-resolved trajectory analyses to avoid cancellation of lateral and rotational components during averaging.

To reflect a common real-world use case of contactless tailgate activation, approximately half of the movements were performed in a loaded condition. In this condition, participants held two objects—a baby car seat and a piece of luggage, one in each hand—while performing the gesture. The remaining movements were performed without an additional load. This design allowed the effects of hand occupancy and associated postural adaptations on gesture execution to be assessed.

Across all participants, gesture types, positions, trajectory variants, legs, and loading conditions, a total of *N* = 6879 segmented movements were included in the final analysis.

### 2.3. IMU Measurement System

**Figure 2:**
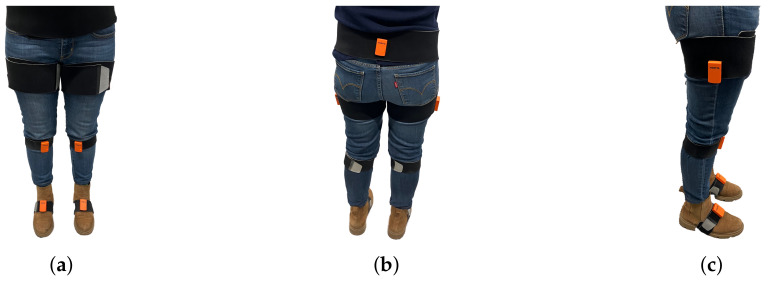
Sensor placement of the seven-sensor lower-body Xsens Awinda configuration. (**a**) Frontal view, (**b**) posterior view, and (**c**) lateral view showing sensors attached to the pelvis, both upper legs, both lower legs, and both feet.

Lower-body motion was recorded using an Xsens Awinda inertial motion-capture system (Movella Inc., Enschede, The Netherlands) in a seven-sensor lower-body configuration. Wireless IMU sensors were attached to the pelvis, both upper legs, both lower legs, and both feet according to the manufacturer-recommended lower-body setup. Sensor placement was checked before each recording to ensure stable attachment to the corresponding body segments. The sensor placement is shown in Figure 2.

Data were recorded at a sampling frequency of 100 Hz—the maximum rate supported by the Xsens Awinda system in the seven-sensor lower-body configuration—and processed in Xsens MVN Analyze using HD reprocessing. The Xsens MVN pipeline estimates three-dimensional segment orientations from the raw inertial sensor signals using a proprietary sensor-fusion algorithm combined with an embedded biomechanical linked-segment body model [17,18]. The sensor-fusion procedure integrates gyroscope, accelerometer, and magnetometer information. In the literature, inertial orientation estimation for such systems is commonly described using Extended Kalman Filter-based approaches, with biomechanical body models used to constrain segment motion [3,17]. HD reprocessing was used for offline processing of the recorded sensor data before exporting segment positions, segment orientations, and joint-level kinematic variables [19]. Based on the resulting segment orientations, lower-body joint angles and segment-level kinematic variables were exported for further analysis.

The exported variables included sensor-fusion-derived Euler joint angles; angular velocities and angular accelerations of the hip, knee, and ankle; and segment positions, velocities, and accelerations of the foot, lower leg, and upper leg. Joint angles were derived from the relative orientations of neighboring body segments within the Xsens biomechanical model. These variables were selected because they describe the spatial and temporal structure of the target movement and can therefore serve as reference information for sensor-system evaluation and future gesture-reproduction tests.

Joint rotations were represented using the ZXY Euler sequence and decomposed into flexion/extension, abduction/adduction, and internal/external rotation. Accordingly, nine lower-limb joint degrees of freedom (DoFs) were analyzed for each movement.

At the beginning of each participant’s measurement session, a Xsens calibration was performed using the standard N-pose procedure according to the manufacturer’s instructions [18]. The same calibration was used for all subsequent recordings of that participant, and a recalibration was performed only if a sensor visibly shifted or detached during the session. The MVN software rated each calibration, and this rating was used as the acceptance criterion. A rating of “good” was accepted directly, and a rating of “acceptable” was additionally verified by a visual plausibility check, in which the participant extended each leg in the anterior, posterior, and both lateral directions and the calibration was accepted only if the segment poses shown in the software visualization matched the executed movements. A rating of “poor” was rejected. Poor ratings were rare and typically caused by magnetometer interference. Following the manufacturer recommendation that the normalized three-axis magnetic norm should not deviate from unity by more than ten percent [20], such cases were identified from the live magnetic-field plots and the calibration was repeated at a different location within the laboratory.

The Xsens system computes orientation with a sensor-fusion filter that continuously corrects the accumulating gyroscope drift using the gravity vector from the accelerometer and the Earth’s magnetic north vector from the magnetometer, yielding a drift-free absolute orientation estimate [17]. The validity of the N-pose calibration therefore depends on the sensors remaining in place rather than on the elapsed time since calibration. The manufacturer specifies a dynamic orientation accuracy of $0.75^{\circ }$ RMS for roll and pitch and $1.5^{\circ }$ RMS for heading [17], and magnetic disturbances predominantly affect the heading estimate rather than the gravity-referenced roll and pitch. For lower-limb joint angles specifically, the concurrent validity of Xsens MVN against an optical reference system has been reported previously, with particularly good agreement for the dominant sagittal-plane angles [3].

### 2.4. Data Acquisition and Preprocessing

Each recording sequence consisted of a continuous IMU recording of multiple consecutive gesture executions rather than isolated single-movement trials. A typical recording sequence contained eight individual movements and lasted approximately 30–45 s. These continuous raw sequences were subsequently segmented into individual kick or wipe executions before further analysis. Segmentation was based on the Euclidean norm of the Cartesian foot-segment position because the executing foot represented the distal segment with the largest spatial excursion during both gesture types. For each original sampling time point $t_{i}$, with a sampling interval of Δt=0.01 s corresponding to the 100 Hz recording frequency, the position-norm $r(t_{i})$ was computed from the foot-segment coordinates $x(t_{i})$, $y(t_{i})$, and $z(t_{i})$ as

(2) $$r\left(t_{i}\right)=\sqrt{x{\left(t_{i}\right)}^{2}+y{\left(t_{i}\right)}^{2}+z{\left(t_{i}\right)}^{2}}.$$

The position-norm $r(t_{i})$ was baseline-corrected, smoothed using a moving-average filter with a window length of 11 samples, and normalized using min–max normalization [0, 1]. Movement candidates were detected as local maxima of the normalized signal using an adaptive threshold *s*:

(3) $$s=\mu_{r}+\sigma_{r},$$

where $\mu_{r}$ and $\sigma_{r}$ denote the mean and standard deviation of the normalized position-norm $r(t_{i})$ within the corresponding recording sequence. The factor of one standard deviation was selected empirically during pilot analyses to balance sensitivity for low-amplitude executions against rejection of baseline fluctuations between consecutive gestures. The detected maxima served as temporal anchor and center points for individual gesture executions, as they represent the point where the foot is farthest underneath the vehicle’s bumper. Around each anchor point, movement onset and offset were determined on the baseline-corrected position-norm signal, yielding one raw movement segment per detected execution. These raw segments were then used for the subsequent filtering, derivation of angular velocities and accelerations, and time normalization. The preprocessing pipeline transformed continuous wearable IMU recordings into movement-level kinematic profiles that can be compared across users and used as human reference data for sensor-system evaluation.

The segmentation was verified by visually inspecting a plot of each segmented movement. Segmentation errors were rare and occurred mainly when a participant performed an additional intermediate step between gestures, which could trigger an additional cut in the position-norm signal.

Joint-angle time series were filtered using a two-stage smoothing procedure based on established biomechanical signal-processing principles [21]. First, an individualized low-pass cutoff frequency was determined for each segmented movement. For each DoF, the signal was filtered with a fourth-order zero-phase Butterworth filter using candidate cutoff frequencies from 2 to 12 Hz, in increments of 0.2 Hz. For each candidate frequency, the root-mean-square residual between the filtered and unfiltered signal was calculated. The optimal cutoff frequency for each DoF was identified as the point of maximum curvature of the residual curve, computed from the second numerical derivative, following the rationale of residual-based cutoff-frequency selection in movement analysis [21]. A movement-specific global cutoff frequency was then calculated as the mean of the nine DoF-specific cutoff frequencies and rounded to the nearest 0.5 Hz. The mean was preferred over the maximum to prevent single high-frequency artifacts in individual DoFs, particularly in low-amplitude non-sagittal rotations, from inflating the global cutoff and reducing smoothing of the dominant sagittal signals. The joint-angle signals of the corresponding movement were subsequently filtered using this global cutoff frequency.

Second, the filtered joint-angle time series were smoothed using a Savitzky–Golay filter with a window length of 11 samples and a polynomial order of 3 [22]. Angular velocities and angular accelerations were derived from the local polynomial representation of the Savitzky–Golay filter. This approach was used to obtain smoothed derivative estimates while limiting noise amplification compared with direct differentiation of the raw joint-angle signals. The individualized cutoff-frequency approach was selected because the dataset contained both comparatively fast kick gestures and slower wipe gestures performed by participants across a broad age range. A fixed cutoff frequency would therefore have been less suitable for preserving movement-specific signal characteristics.

For time-normalized analyses, each segmented movement was resampled from its original duration-dependent time vector to a common normalized time grid representing 0–100% of the movement cycle. The normalized grid consisted of 100 equally spaced points between 0 and 100% of the gesture cycle. This procedure transformed each segmented movement into a time-normalized trajectory with identical length while preserving the relative temporal structure of the movement. Time normalization was used for the comparison of joint-angle trajectories across participants, gesture types, and movement directions, as well as for the principal component analysis described below. For wipe gestures, clockwise and counterclockwise executions were kept separate in time-resolved analyses to avoid cancellation of lateral and rotational components.

A predefined non-iterative outlier procedure was applied to remove technical recording artifacts and rare extreme executions from the derived summary metrics. For each analysis table, including RoM, segment velocities, segment accelerations, angular velocities, and angular accelerations, outlier limits were computed from the corresponding primary metrics over the complete dataset using the conventional 1.5-IQR rule for boxplot-based outlier detection [23]. The interquartile range (IQR) was defined as

(4) $$\mathrm{IQR}=Q_{0.75}-Q_{0.25},$$

where $Q_{0.25}$ and $Q_{0.75}$ denote the 25th and 75th percentiles of the corresponding metric, respectively. Values outside the interval

(5) $$\left[Q_{0.25}-1.5\cdot \mathrm{IQR},\;Q_{0.75}+1.5\cdot \mathrm{IQR}\right]$$

were excluded. The thresholds were computed once on the complete dataset and were not recalculated separately for gesture types, positions, loading conditions, or demographic subgroups. This ensured that the filtering procedure was applied consistently across all subsequent analyses.

### 2.5. Kinematic Variables and Reference Profiles

In this study, a kinematic reference profile denotes the mean and the variability of each joint- and segment-level variable across all observed executions—reported as standard deviations in the tables and as medians, quartiles, and interquartile ranges in the boxplots—and is intended as a benchmark range for sensor evaluation and future comparison. At the joint level, the analyzed variables comprised RoM, angular velocity, and angular acceleration for the nine lower-limb DoFs described above. RoM was computed as the difference between the maximum and minimum joint angle within each segmented movement. Angular velocities and angular accelerations were derived from the smoothed joint-angle trajectories as described in the preprocessing section.

At the segment level, position, velocity, and acceleration profiles were analyzed for the foot, lower leg, and upper leg of the executing leg. Segment velocities and accelerations were evaluated both as resultant three-dimensional magnitudes and as radial components relative to the vehicle-facing direction. The resultant magnitude describes the overall segment motion independent of direction. The radial component was defined as the projection of the segment velocity and acceleration vectors onto the vehicle longitudinal axis established during calibration—that is, the horizontal axis aligned with the vehicle-facing direction. This axis was used as an approximation of the dominant sensor-target direction of a bumper-integrated rear radar, while the exact mapping to a specific sensor depends on its mounting position and beam geometry. This distinction was included because contactless tailgate activation systems observe the same human movement from a fixed vehicle-integrated sensor perspective, and the observable signal depends on the spatial relationship between the movement trajectory and the sensing direction [10,11].

For each segmented movement, summary metrics were computed from the time-resolved joint and segment profiles. These metrics included movement duration, joint RoM, maximum and mean absolute angular velocity, maximum and mean absolute angular acceleration, maximum segment velocity, and maximum segment acceleration. The resulting values were used to construct gesture-specific reference profiles for kick and wipe movements, and to compare variability across gesture type, position, loading condition, and demographic subgroup.

### 2.6. Principal Component Analysis

Principal component analysis (PCA) was used to identify dominant coupled joint-motion patterns and to quantify the variance structure of the segmented gesture executions [24,25]. The PCA was performed on the time-normalized joint-angle trajectories and was calculated separately for kick and wipe movements to allow gesture-specific movement structures to be compared. The input matrix was constructed so that each row represented one time point of one segmented movement and each column represented one of the nine analyzed lower-limb DoFs.

Before PCA, the joint-angle data were mean-centered. No variance standardization was applied because all variables were expressed in the same physical unit (degrees), and because the relative amplitude of each DoF was considered physiologically meaningful for the movement characterization. The PCA was therefore based on the covariance matrix rather than the correlation matrix. This approach preserved the contribution of large-amplitude joint motions to the overall movement variance.

The proportion of variance explained (PVE) by the *k*-th principal component was computed as

(6) $${\mathrm{PVE}}_{k}=\frac{\lambda_{k}}{\sum_{j=1}^{9}\lambda_{j}},$$

where $\lambda_{k}$ denotes the *k*-th eigenvalue of the covariance matrix, ordered in descending magnitude. The corresponding eigenvectors $v_{k}$ contained the loadings $v_{jk}$, which describe the contribution of DoF *j* to principal component *k*. Loadings with an absolute value of $|v_{jk}|\geq 0.30$ were treated as dominant contributors for qualitative interpretation of the corresponding component, following common interpretative conventions for component and factor loadings [26].

To quantify the aggregated contribution of each DoF across all principal components, a variance-weighted loading measure was computed as

(7) $$w_{j}=\sum_{k=1}^{9}{\mathrm{PVE}}_{k}\cdot v_{jk}^{2},$$

where $w_{j}$ denotes the variance-weighted contribution of DoF *j*. This measure summarizes how strongly each DoF contributed to the total joint-angle variability while accounting for the variance explained by each component.

Two complementary PCAs were performed to assess the robustness of the identified movement structures. First, gesture-specific PCAs were repeated after excluding the three sagittal flexion/extension DoFs of the hip, knee, and ankle. This analysis was used to evaluate how strongly the overall component structure depended on the dominant sagittal joint motions. Second, PCA was performed separately for loaded and unloaded executions to assess whether hand occupancy altered the main joint-motion structure.

### 2.7. Statistical Analysis

The statistical analysis was primarily descriptive, consistent with the aim of deriving kinematic reference profiles rather than testing subgroup hypotheses. To confirm the central finding—that kick and wipe gestures share a common sagittal basis but differ in their non-sagittal components—one confirmatory comparison was performed at the participant level. For each of the nine DoFs, the per-participant mean RoM was compared between gesture types over the n=56 participants. A paired test was required because every participant performed both gestures, and the two-sided Wilcoxon signed-rank test was used because it does not assume normally distributed differences, making it appropriate for the small, lower-bounded non-sagittal RoM values. The participant-level mean was used as the unit of analysis, so that each participant contributed one paired observation and repeated executions did not inflate the sample size. Effect sizes were reported as the matched-pairs rank-biserial correlation, and the nine *p*-values were Holm-corrected. All other comparisons were interpreted descriptively, because the study was not powered for subgroup inference.

Continuous variables were summarized using the mean and standard deviation where aggregate reference values were required. Distributional characteristics were additionally described using medians, quartiles, and boxplot-based visualizations where appropriate. Gesture-specific reference profiles were reported separately for kick and wipe movements. Additional descriptive comparisons were performed across loading condition, starting position, and demographic subgroup.

Because multiple repeated gesture executions were recorded per participant, the individual segmented movement was used as the primary unit for movement-level reference profiles. This choice was consistent with the aim of describing the full range of executed gestures, including within-subject variability, as encountered by a sensor system in practical use. Formal mixed-effects modeling with participant as a random effect was not applied because the present study did not test population-level effects, and because the resulting reference profiles are intended to represent the distribution of observed movement executions rather than to estimate per-participant typical values. Subgroup results, particularly those for age and sex, were interpreted descriptively because the subgroup sizes were unbalanced and the study was not powered for formal demographic inference.

## 3. Results

### 3.1. Dataset and Preprocessing Characteristics

A total of N=6879 segmented lower-limb gesture executions from n=56 participants were included in the final analysis. The dataset comprised $N_{k}=3523$ kick movements and $N_{w}=3356$ wipe movements. Of these, 3701 were performed without an additional load and 3178 in the loaded condition. The segmented movements covered both executing legs, multiple starting positions, trajectory variants, and loaded as well as unloaded executions. The complete data flow from recorded participants to the final movement counts is summarized in Table 3.

The individualized cutoff frequencies determined by the preprocessing pipeline were low and consistent across the dataset, with a mean value of 2.69±0.27 Hz and a median value of 2.5 Hz. This indicates that the recorded gestures were predominantly low-frequency, continuous, and submaximal movements.

Application of the movement-level 1.5-IQR criterion retained 6118 of the 6879 segmented movements, corresponding to 88.9% of the original dataset. A total of 761 movements were excluded because at least one of the nine joint range-of-motion variables was outside its respective IQR-based limits.

### 3.2. Temporal Characteristics

The segmented movements showed similar durations for both gesture types. Kick movements had a mean duration of 2.16±0.15 s, whereas wipe movements had a mean duration of 2.23±0.22 s. The absolute difference between the mean gesture durations was 0.07 s.

### 3.3. Joint Range of Motion

**Figure 3:**
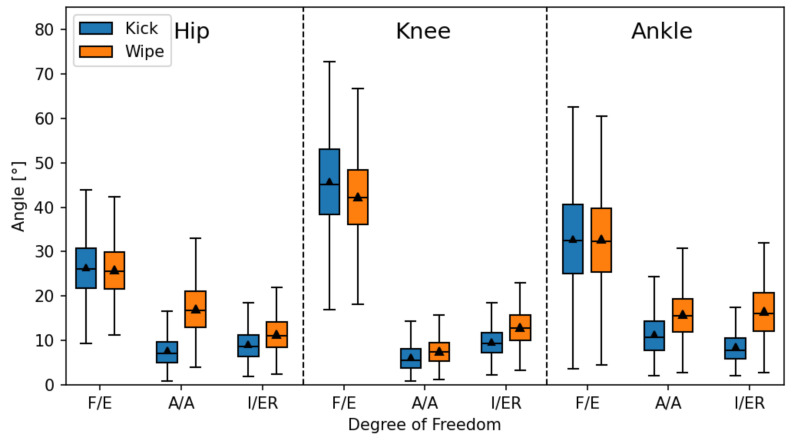
Joint RoM for kick and wipe movements. Values are shown for the analyzed lower-limb DoFs. Abbreviations: F/E, flexion/extension; A/A, abduction/adduction; I/ER, internal/external rotation.

The joint RoM differed between gesture types and DoFs, as shown in Figure 3. For kick movements, the largest RoM occurred in knee flexion/extension, followed by ankle flexion/extension and hip flexion/extension. Knee flexion/extension reached $45.9\pm 10.9^{\circ }$, ankle flexion/extension reached $32.8\pm 10.9^{\circ }$, and hip flexion/extension reached $26.5\pm 6.3^{\circ }$.

For wipe movements, the largest sagittal RoM values were also observed in knee flexion/extension, ankle flexion/extension, and hip flexion/extension. Knee flexion/extension reached $42.3\pm 9.0^{\circ }$, ankle flexion/extension reached $32.8\pm 10.0^{\circ }$, and hip flexion/extension reached $25.9\pm 6.0^{\circ }$.

Differences between gesture types were most apparent in the non-sagittal DoFs. Hip abduction/adduction reached $7.7\pm 3.6^{\circ }$ for kick movements and $17.2\pm 5.6^{\circ }$ for wipe movements. Ankle internal/external rotation reached $8.7\pm 3.8^{\circ }$ for kick movements and $16.6\pm 6.1^{\circ }$ for wipe movements. The remaining non-sagittal DoFs showed smaller differences between gesture types.

### 3.4. Principal Component Analysis

**Figure 4:**
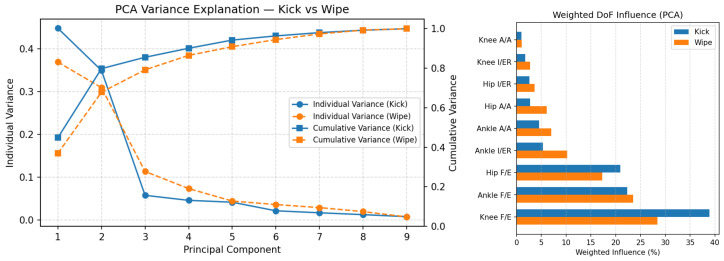
Principal component analysis of wearable IMU-derived joint-angle trajectories. (**Left**) Individual and cumulative proportion of variance explained by the principal components for kick and wipe movements. (**Right**) Variance-weighted DoF contributions $w_{j}$ across all principal components. Abbreviations: F/E, flexion/extension; A/A, abduction/adduction; I/ER, internal/external rotation.

The gesture-specific PCA revealed different variance structures for kick and wipe movements. The explained variance structure and the variance-weighted DoF contributions are shown in Figure 4. The corresponding loading matrices for the first four principal components are reported in Table 4.

For kick movements, PC1 accounted for 44.8% of the total variance and PC2 accounted for 35.0%. Together, PC1 and PC2 explained 79.8% of the total variance. PC3 contributed 5.7% and PC4 contributed 4.6%. The largest loadings in PC1 were observed for knee flexion/extension and hip flexion/extension. PC2 showed the largest loading for ankle flexion/extension, with additional contributions from knee flexion/extension and hip flexion/extension. The variance-weighted DoF contributions were highest for knee flexion/extension at 38.9%, ankle flexion/extension at 22.3%, and hip flexion/extension at 21.0%. Together, these three sagittal DoFs accounted for 82.1% of the variance-weighted total movement variability.

For wipe movements, PC1 accounted for 36.9% of the total variance and PC2 accounted for 30.9%. Together, PC1 and PC2 explained 67.8% of the total variance. PC3 contributed 11.3% and PC4 contributed 7.3%. The largest loadings in PC1 were observed for ankle flexion/extension and hip flexion/extension. PC2 was dominated by knee flexion/extension. PC3 showed the largest loading for ankle internal/external rotation, and PC4 showed the largest loading for hip abduction/adduction. The variance-weighted DoF contributions were highest for knee flexion/extension at 28.4%, ankle flexion/extension at 23.5%, and hip flexion/extension at 17.3%. Ankle internal/external rotation contributed 10.2%, and hip abduction/adduction contributed 6.1%.

The complementary PCAs showed that the variance structure changed when the sagittal flexion/extension DoFs were excluded. In this analysis, the cumulative variance explained by the first three components was 78.8% for kick movements and 80.6% for wipe movements.

In the loading-condition analysis, the PCA structure was similar between loaded and unloaded executions. The first two components explained 74.2% of the total variance under loaded conditions and 73.1% under unloaded conditions. The first three components explained 82.3% under loaded conditions and 81.9% under unloaded conditions. The ranking of the variance-weighted DoF contributions was identical in both conditions, with knee flexion/extension, ankle flexion/extension, and hip flexion/extension as the three largest contributors.

### 3.5. Time-Normalized Gesture Profiles and Phase Structure

**Figure 5:**
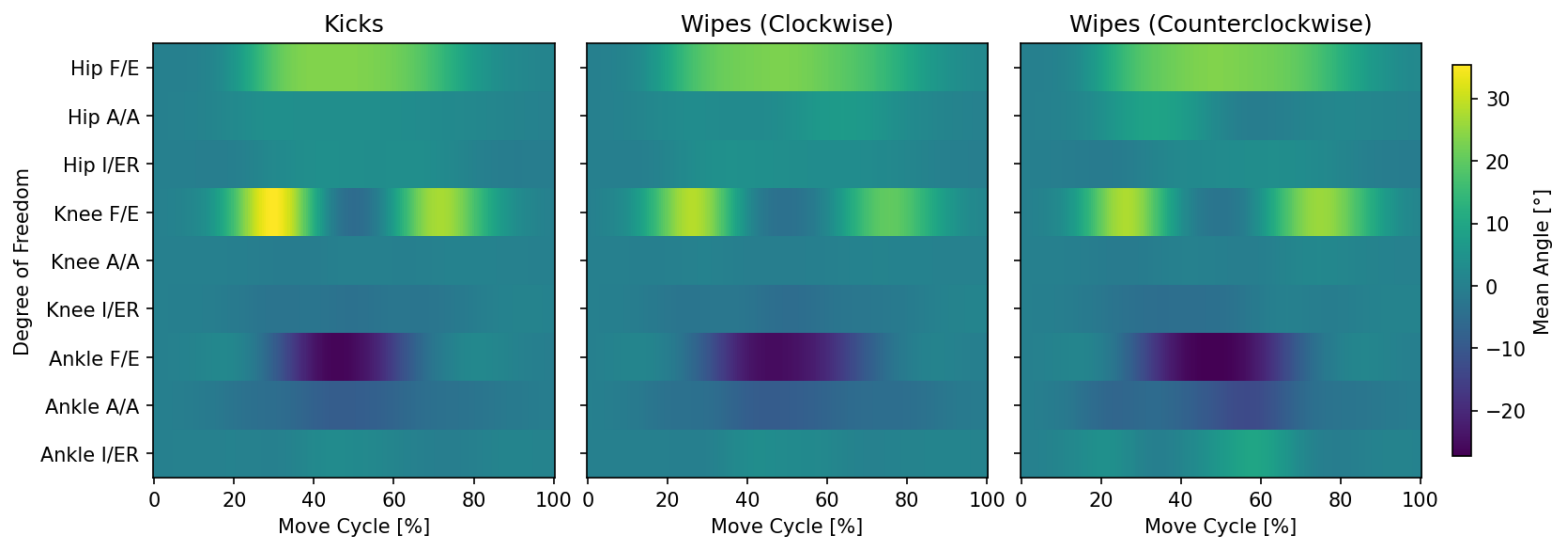
Time-normalized joint-angle profiles for kick movements, and for clockwise and counterclockwise wipe movements. Values are shown over the normalized movement cycle from 0 to 100%. Abbreviations: F/E, flexion/extension; A/A, abduction/adduction; I/ER, internal/external rotation.

The time-normalized joint-angle profiles showed a consistent temporal structure across both gesture types (Figure 5). For kick movements, the largest time-dependent changes occurred in the sagittal DoFs. Knee flexion/extension increased during the first part of the movement, decreased toward the middle of the movement cycle, and increased again during the return phase. Hip flexion/extension and ankle flexion/extension showed their largest excursions around the middle of the movement cycle.

For wipe movements, the sagittal DoFs showed a temporal pattern similar to the kick movements. Additional time-dependent changes were observed in non-sagittal DoFs. Clockwise and counterclockwise wipe movements showed opposite signs in the lateral and rotational components, which is reflected by the mirrored color patterns in the hip abduction/adduction and ankle internal/external rotation. Therefore, both movement directions are displayed separately in Figure 5.

**Figure 6:**
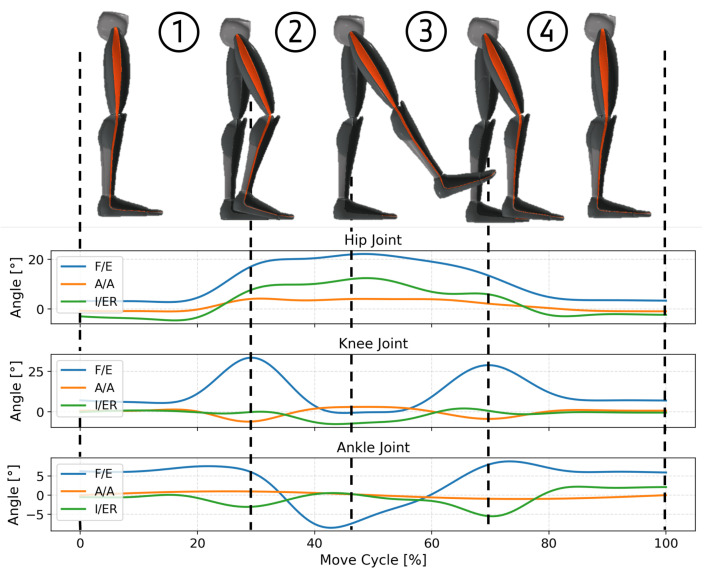
Representative four-phase temporal structure of a kick movement based on sagittal joint-angle profiles.

The sagittal joint-angle profiles exhibited a characteristic four-phase temporal structure consisting of an initial forward movement, a transition toward the most extended knee position, an initial return phase, and a final return to the starting stance. A representative example is shown in Figure 6 for a kick movement.

### 3.6. Joint-Angle Velocities and Accelerations

**Figure 7:**
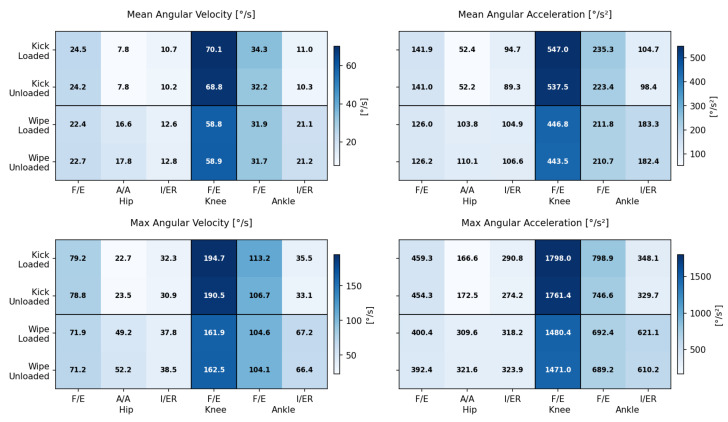
Angular velocities and angular accelerations by gesture type, loading condition, and DoF. (**Upper row**) mean absolute values over the movement cycle. (**Lower row**) maximum values per segmented movement. Abbreviations: F/E, flexion/extension; A/A, abduction/adduction; I/ER, internal/external rotation.

Joint-angle velocities and accelerations showed different magnitudes across gesture types, loading conditions, and DoFs (Figure 7). For kick movements, the highest peak angular velocities were observed in the sagittal DoFs. Knee flexion/extension reached 194.7°/s under loaded conditions and 190.5°/s under unloaded conditions. Ankle flexion/extension reached 113.2°/s and 106.7°/s, and hip flexion/extension reached 79.2°/s and 78.8°/s, under loaded and unloaded conditions, respectively.

The corresponding mean absolute angular velocities were lower than the peak values. For kick movements, knee flexion/extension reached $70.1^{\circ }$/s under loaded conditions and $68.8^{\circ }$/s under unloaded conditions. Ankle flexion/extension reached $34.3^{\circ }$/s and $32.2^{\circ }$/s, and hip flexion/extension reached $24.5^{\circ }$/s and $24.2^{\circ }$/s, under loaded and unloaded conditions, respectively.

For wipe movements, knee flexion/extension reached peak angular velocities of $161.9^{\circ }$/s under loaded conditions and $162.5^{\circ }$/s under unloaded conditions. Hip abduction/adduction reached $49.2^{\circ }$/s and $52.2^{\circ }$/s, and ankle internal/external rotation reached $67.2^{\circ }$/s and $66.4^{\circ }$/s, under loaded and unloaded conditions, respectively. The corresponding mean absolute angular velocities for hip abduction/adduction were $16.6^{\circ }$/s and $17.8^{\circ }$/s. For ankle internal/external rotation, the mean absolute angular velocities were $21.1^{\circ }$/s and $21.2^{\circ }$/s under loaded and unloaded conditions, respectively.

Angular accelerations followed the same overall ordering of DoFs. For kick movements, knee flexion/extension showed the highest peak angular accelerations, reaching $1798^{\circ }$/$s^{2}$ under loaded conditions and $1761^{\circ }$/$s^{2}$ under unloaded conditions. The corresponding mean absolute angular accelerations were $547^{\circ }$/$s^{2}$ and $538^{\circ }$/$s^{2}$. Across the reported angular velocity and acceleration metrics, the differences between loaded and unloaded conditions were small relative to the differences between gesture-specific DoFs.

### 3.7. Segment Velocities and Accelerations

**Figure 8:**
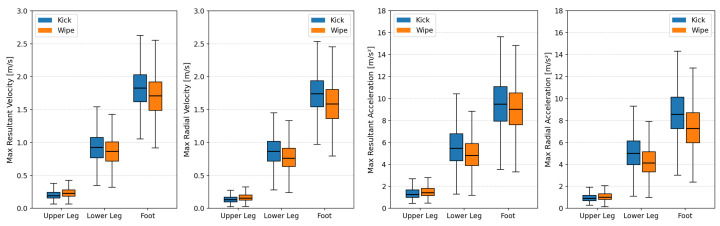
Maximum segment velocities and accelerations for foot, lower leg, and upper leg during kick and wipe movements. (**Left**) Maximum segment velocities. (**Right**) Maximum segment accelerations. For each variable, resultant magnitudes and radial components are shown.

Segment velocities and accelerations differed between segments, gesture types, and vector components (Figure 8). For both gesture types, the highest values were observed at the foot, followed by the lower leg and upper leg.

For kick movements, the foot reached a mean maximum resultant velocity of 1.83m/s, the lower leg reached 0.93m/s, and the upper leg reached 0.21m/s. The corresponding mean maximum radial velocities were 1.75m/s for the foot, 0.87m/s for the lower leg, and 0.14m/s for the upper leg.

For wipe movements, the foot reached a mean maximum resultant velocity of 1.71m/s, the lower leg reached 0.87m/s, and the upper leg reached 0.24m/s. The corresponding mean maximum radial velocities were 1.59m/s for the foot, 0.78m/s for the lower leg, and 0.17m/s for the upper leg.

The segment accelerations showed the same segment ordering. The mean maximum resultant foot acceleration was $9.6\;m/s^{2}$ for kick movements and $9.1\;m/s^{2}$ for wipe movements. The mean maximum radial foot acceleration was $8.8\;m/s^{2}$ for kick movements and $7.4\;m/s^{2}$ for wipe movements. The lower leg and upper leg accelerations were lower than foot accelerations for both gesture types.

### 3.8. Position-Dependent Variability

**Figure 9:**
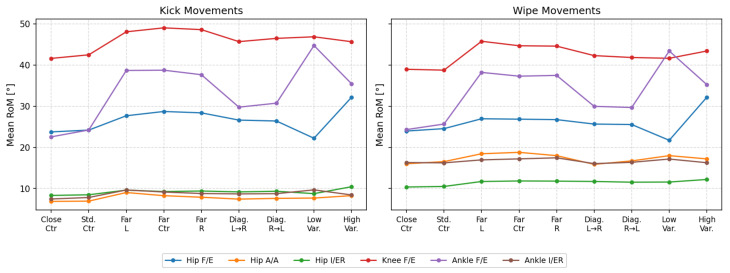
Joint RoM by starting position for kick and wipe movements. Values are shown for selected lower-limb DoFs. Abbreviations: F/E, flexion/extension; A/A, abduction/adduction; I/ER, internal/external rotation; Ctr, center; Std, standard; L, left; R, right; Diag, diagonal; Var, variant.

Position-dependent differences in joint RoM are shown in Figure 9. The overall RoM pattern was similar across starting positions for both gesture types. The largest position-dependent differences were observed in the sagittal DoFs.

For knee flexion/extension, RoM increased with increasing distance from the vehicle. At the close center position, knee flexion/extension reached $41.6^{\circ }$ for kick movements and $39.0^{\circ }$ for wipe movements. At the standard center position, the corresponding values were $42.5^{\circ }$ and $38.8^{\circ }$. Across the far positions, the mean knee flexion/extension reached $48.6^{\circ }$ for kick movements and $45.0^{\circ }$ for wipe movements.

Ankle flexion/extension also showed position-dependent differences. For kick movements, ankle flexion/extension increased from $22.5^{\circ }$ at the close center position to an average of $38.3^{\circ }$ across the far positions. The diagonal positions showed intermediate values between the close, standard, and far positions.

The instructed high- and low-trajectory variants extended the observed RoM range. The high variant showed the largest hip flexion/extension values, with $32.2^{\circ }$ for kick movements and $32.2^{\circ }$ for wipe movements. The low variant showed the largest ankle flexion/extension values, with $44.7^{\circ }$ for kick movements and $43.4^{\circ }$ for wipe movements.

The non-sagittal differences between kick and wipe movements were visible across positions. Hip abduction/adduction and ankle internal/external rotation were higher for wipe movements than for kick movements at the analyzed positions.

### 3.9. Loading-Condition Comparison

Descriptive differences in joint RoM between the loaded and unloaded conditions are reported in Table 5, complementing the angular and segment velocity results already reported by loading condition in Section 3.6 and Section 3.7. In the loaded condition, participants held a baby car seat of 4.30 kg in one hand and a small piece of luggage of 2.45 kg in the other hand while performing the gesture. All absolute differences between the loaded and unloaded conditions were below one degree, and the largest relative difference was 6.4% in the small knee internal/external rotation component. The differences were therefore small relative to the gesture-specific differences between kick and wipe movements, and the ranking of the dominant DoFs was preserved.

### 3.10. Demographic Variability

Descriptive RoM values by sex and age group are reported in Table 6. These subgroup results were not subjected to inferential statistical testing and are therefore reported as descriptive observations only. Because the study was designed to derive kinematic reference profiles rather than to test demographic effects, and because the subgroup sizes were unbalanced, particularly in the older age groups, the values should not be interpreted as statistically significant group differences.

Across age groups, the lowest descriptive knee flexion/extension values were observed in the 70+ group, with $38.5^{\circ }$ for kick movements and $34.7^{\circ }$ for wipe movements. The remaining age groups showed knee flexion/extension values ranging from $43.9^{\circ }$ to $47.5^{\circ }$ for kick movements and from $41.0^{\circ }$ to $43.4^{\circ }$ for wipe movements. Because the 70+ group comprised only two participants, this observation should be interpreted cautiously and should not be generalized as an age-related effect.

### 3.11. Confirmatory Comparison of Gesture-Specific Degrees of Freedom

The participant-level Wilcoxon signed-rank comparison confirmed the gesture-specific structure observed in the descriptive profiles. The full results for all nine DoFs are reported in Table 7. The two non-sagittal DoFs that characterize the wipe gesture differed strongly between gesture types, with larger values for wipe movements in hip abduction/adduction and ankle internal/external rotation (both z=−6.51, p<0.001, $r_{rb}=1.00$). In both cases, every participant showed a larger RoM for the wipe gesture than for the kick gesture. In contrast, the two dominant sagittal DoFs behaved as expected for a shared movement basis. Hip flexion/extension did not differ significantly between gesture types (z=−1.79, $p_{\mathrm{Holm}}=0.148$), and ankle flexion/extension showed no relevant difference (z=−0.05, $p_{\mathrm{Holm}}=0.961$). Knee flexion/extension differed significantly with a moderate effect size and a larger RoM for kick movements (z=−3.64, p<0.001, $r_{rb}=-0.56$), consistent with the descriptive RoM values. The remaining non-sagittal DoFs also differed significantly, several of them with large effect sizes, but their absolute RoM amplitudes were small compared with the hip abduction/adduction and ankle internal/external rotation components and are therefore not interpreted as primary gesture-distinguishing features. This confirmatory analysis supports the interpretation that kick and wipe gestures share a common sagittal structure and are primarily distinguished by their non-sagittal hip and ankle components.

## 4. Discussion

### 4.1. Main Findings

This study provides wearable IMU-derived kinematic reference profiles for lower-limb kick and wipe gestures used in contactless automotive tailgate activation. The main finding is that both gesture types share a common sagittal baseline structure dominated by knee flexion/extension, ankle flexion/extension, and hip flexion/extension. This shared structure was consistently observed in the RoM analysis, angular velocity and acceleration profiles, time-normalized trajectories, and PCA results.

Despite this common sagittal structure, kick and wipe gestures differed substantially in their secondary DoFs. Wipe movements showed markedly higher hip abduction/adduction and ankle internal/external rotation than kick movements, both in angular amplitude and dynamic metrics. These differences were also reflected in the PCA, where wipe movements showed additional variance contributions from lateral and rotational components. This gesture-specific difference in the non-sagittal components was statistically confirmed on a participant level (both p<0.001, rank-biserial correlation $r_{rb}=1.00$). The wipe gesture therefore represents a distinct lower-limb HMI movement pattern rather than a simple variant of a sagittal kick gesture.

A second observation is that the core kinematic structure appeared consistent across loading conditions and most demographic subgroups in the descriptive analysis. This interpretation is based on descriptive comparisons only and was not formally tested, because the study was not powered for subgroup inference. Holding objects with both hands had only a minor influence on the joint-level movement structure and did not alter the ranking of the dominant DoFs. Similarly, sex-related differences were small compared with the gesture-specific differences between kick and wipe movements. A lower sagittal RoM was observed in the oldest age group, but this subgroup contained only two participants and should therefore be interpreted descriptively.

Finally, the segment-level results showed a clear proximal-to-distal dynamic hierarchy, with the foot reaching the highest velocities and accelerations. The difference between resultant and radial foot velocity was larger for wipe movements than for kick movements, indicating that wipe gestures contain a larger non-radial movement component.

### 4.2. Gesture-Specific Kinematic Structure

The observed movement structure confirms that contactless automotive tailgate gestures form a distinct class of lower-limb movement. Although the term “kick” is commonly used for the forward-directed gesture, the measured kinematics differed substantially from sport-specific kicking movements [13,14,15]. The longer mean gesture durations of approximately 2.2 s and the peak knee flexion/extension velocities of roughly 200°/s reflect the different task constraints of submaximal, contact-free HMI execution compared with performance-oriented sport kicks.

Kick and wipe gestures nevertheless shared a common sagittal movement basis. Knee flexion/extension, ankle flexion/extension and hip flexion/extension dominated the RoM profiles, the dynamic variables, and the variance-weighted PCA contributions. This shared structure can be interpreted as the biomechanical requirement of moving the foot toward and away from the rear bumper. The key difference between both gesture types was not the sagittal movement component but the additional lateral and rotational contribution in wipe movements.

These non-sagittal differences were present in the RoM, angular velocity, and PCA results, indicating that the wipe gesture is not merely a slower or less linear variant of the kick gesture. Instead, it represents a distinct interaction movement with a characteristic non-sagittal component. This is relevant for gesture recognition because a feature representation based only on sagittal foot motion would capture the shared movement basis but would not fully represent the gesture-specific structure of wipe movements.

The PCA results support this interpretation at the level of coordinated joint motion. Because the PCA was computed on the covariance matrix without variance standardization, the dominance of the high-amplitude sagittal DoFs in the leading components partly reflects their larger absolute range. The more informative aspect of the component structure is therefore not which DoFs are largest but how they co-vary, and particularly the additional lateral and rotational components that distinguish wipe from kick movements. In kick movements, the first two principal components explained most of the variance and were dominated by sagittal DoFs. In wipe movements, the first two components were also sagittal, but additional variance appeared in components dominated by ankle internal/external rotation and hip abduction/adduction. This broader variance distribution is consistent with the larger execution freedom of a curved wipe trajectory and with the direction-dependent lateral and rotational components observed in the time-normalized joint-angle profiles.

The kinematic profiles also position the analyzed gestures within the broader lower-limb movement landscape. A recent PCA-based kinematic synergy analysis characterized 34 locomotor and whole-body motor tasks, including walking, running, hopping, turning, and sitting-down–standing-up [27]. Discrete HMI gestures such as the kick and wipe analyzed here are not represented in that taxonomy, suggesting that contactless tailgate gestures form a movement class outside the conventional locomotor and whole-body categories. Compared with typical gait reference values of approximately 40° for hip flexion/extension and 60° for knee flexion/extension during the gait cycle [21], the present sagittal excursions of $27^{\circ }$ at the hip and $46^{\circ }$ at the knee for kick gestures, and $26^{\circ }$ and $42^{\circ }$ for wipe gestures, are smaller. This is consistent with the submaximal, non-locomotor nature of the analyzed HMI gestures and supports the interpretation that sport-biomechanical or gait-derived reference data are not directly applicable to this movement class.

### 4.3. Relevance for Wearable Inertial Sensing and Vehicle-Integrated Gesture Recognition

The present study demonstrates how wearable inertial motion capture can be used to derive application-specific human movement reference profiles for sensor-system evaluation. Vehicle-integrated gesture-recognition systems are usually evaluated at the level of sensor signals and classification performance, whereas the underlying human target motion is often treated as an implicitly defined input. The IMU-derived profiles reported here describe this target motion directly at the joint and segment levels. This creates a reference layer between human movement execution and technical sensor response.

This reference layer is relevant because different sensing modalities observe different aspects of the same movement. For radar-based systems in particular, the radial component of the moving target is decisive because Doppler-based measurements are linked to motion along the sensor-target direction [11,28]. The larger difference between resultant and radial foot velocity observed in wipe gestures, compared with kick gestures, indicates a stronger non-radial component due to the curved or lateral foot trajectory. A radar system relying primarily on radial velocity may therefore capture the sagittal approach and retraction components of both gestures but underrepresent the lateral component that differentiates wipes from kicks. This does not imply that radar systems cannot recognize wipe gestures, but it indicates that trajectory-aware, spatial, or multi-feature approaches are particularly relevant for such movements.

The present study complements previous work on smart-trunk or foot-gesture recognition, which has primarily focused on sensor-signal processing and classification performance using radar or dedicated foot-gesture sensors [10,11,12]. The resulting reference profiles define joint-level and segment-level kinematic ranges that a sensing system should be expected to observe under realistic user execution. They can therefore guide the definition of realistic target trajectories for sensor simulation and algorithm development, provide benchmark ranges for evaluating vehicle-integrated sensors, and support the design of repeatable robotic or mechanical test procedures that reproduce human-like gesture execution. In particular, the joint-level ranges and time-normalized trajectories define the input required to drive a robotic leg that reproduces the human gesture, enabling objective and automated kick and wipe executions whose repeatability is independent of individual human variability and, thus, supports standardized and reproducible sensor testing.

The reference profiles are also relevant for potential future camera-based tailgate activation systems. Vision-based gesture-recognition approaches in automotive HMI have already been investigated for in-vehicle interaction, particularly using red, green, and blue (RGB), depth, or time-of-flight (ToF) cameras [29,30]. More generally, camera-based gesture recognition can use image features, depth information, or estimated skeletal joint trajectories as input for classification [31]. If exterior vehicle cameras are used in future systems to observe the user behind the vehicle and infer lower-limb motion through pose estimation or markerless motion-capture methods, the present IMU-derived joint and segment profiles provide an independent kinematic reference. They can support the definition of plausible kick and wipe trajectories, the validation of camera-derived lower-limb joint estimates, and the interpretation of recognition errors caused by occlusion, viewpoint variation, or incomplete visibility of the foot and lower leg.

The position-dependent results further indicate that gesture execution varies systematically with the user position relative to the vehicle. Greater distance from the rear bumper increased sagittal joint excursions, particularly knee flexion/extension and ankle flexion/extension. Therefore, test protocols for contactless tailgate activation should not be limited to a single nominal stance position. Instead, they should include different distances, lateral offsets, and trajectory heights in order to represent realistic interaction variability.

### 4.4. Practical Implications for Sensor System Design and Evaluation

The established kinematic reference profiles provide a quantitative basis for the design and optimization of vehicle-integrated sensing systems. Beyond the biomechanical description, several engineering-relevant observations can be derived from the data, which should be interpreted as biomechanical guidance rather than as validated design parameters.

Current radar-based systems often rely on Doppler signal strength to trigger tailgate activation. Our results show that the foot segment consistently exhibits the highest resultant and radial velocities among all analyzed segments. This suggests that sensor sensitivity tuned to the Doppler signatures of the foot’s radial velocity components is likely to capture the dominant motion of both gesture types. The reported mean radial velocities and their standard deviations may serve as a starting point for defining candidate detection thresholds that remain sensitive to submaximal or slow executions—for example, by older users—while leaving margin against environmental noise. Sensor-specific validation against vehicle-integrated recordings is required before such thresholds can be applied operationally.

The temporal analysis revealed a mean gesture duration of approximately 2.2 s for both kick and wipe movements. This duration informs the configuration of sliding-window classification algorithms such as Long Short-Term Memory (LSTM) or Convolutional Neural Network (CNN). Window lengths in the range of 2.5 to 3.0 s appear adequate to cover the full intentional gesture in the present dataset. The observation that the kinematic reversal point occurs at approximately 50–60% of the gesture cycle further suggests that early-stage classification may be feasible before the leg returns to the starting position, although the latency benefit of such an approach would require dedicated classifier evaluation.

The adaptive filtering analysis identified a mean cutoff frequency of approximately 2.7 Hz for the recorded IMU-derived joint-angle trajectories. This low-frequency characteristic suggests that the underlying human gesture motion is dominated by relatively slow, continuous components. For vehicle-integrated sensing systems, this finding may inform the selection of candidate temporal windows and filter ranges. However, sensor-specific validation remains necessary because radar, capacitive, and other vehicle-integrated sensors observe different physical quantities than wearable IMUs.

The PCA results may also inform feature-engineering hypotheses for future multimodal gesture-recognition studies. For kick gestures, the concentration of variability in the sagittal plane suggests that longitudinal and vertical motion components are likely to be informative. In contrast, the broader lateral and rotational contributions found in wipe gestures indicate that multi-planar feature sets may be required for robust wipe detection.

### 4.5. Limitations and Future Work

Several limitations should be considered when interpreting the results of this study. First, data collection was performed using a single vehicle model and a fixed rear-bumper geometry. Although the experimental setup was designed to represent realistic contactless tailgate activation, vehicle-specific factors such as bumper height, bumper shape, and sensor integration area may influence gesture execution. The resulting reference profiles should therefore be interpreted as application-specific for the investigated vehicle geometry. Future studies should include additional vehicle classes, including compact vehicles, sedans, and vehicles with lower or higher bumper geometries, to determine how strongly vehicle geometry affects lower-limb gesture kinematics.

Second, the measurements were acquired under controlled experimental conditions. The predefined starting positions, trajectory variants, and loading condition were selected to cover practically relevant use cases, but real-world environments may introduce additional variability. Examples include uneven ground, limited space behind the vehicle, adverse weather, different footwear, poor lighting for vision-based systems, and spontaneous user behavior. Future work should therefore validate the reference profiles in more naturalistic outdoor scenarios and during unscripted vehicle interaction.

Third, this study used a wearable IMU-based motion-capture system rather than an optical reference system. This choice was appropriate for the application context because camera-based marker tracking around the rear bumper can be affected by marker occlusion and restricted installation geometry. However, IMU-derived joint angles can be affected by sensor placement, calibration quality, soft-tissue motion, magnetic disturbances, and sensor-fusion assumptions [7,32,33]. These error sources do not affect all degrees of freedom equally. Magnetic disturbances act primarily on the heading estimate [17] and, therefore, most strongly on the internal/external rotation components, while the dominant sagittal flexion/extension is gravity-referenced and less sensitive. Soft-tissue motion and small placement errors also represent a larger fraction of the observed amplitude for the small non-sagittal rotations than for the large sagittal excursions. The absolute magnitudes of the smaller hip abduction/adduction and ankle internal/external rotation components should therefore be interpreted with more caution than the dominant sagittal components. Importantly, the central finding that wipe gestures contain a substantially larger non-sagittal contribution than kick gestures does not depend on this absolute precision, because the gesture-related differences are large—for example, an approximately twofold difference in ankle internal/external rotation RoM between kick and wipe movements. The dominant sagittal DoFs are expected to be the most robust overall.

Fourth, the statistical analyses were primarily descriptive. A participant-level confirmatory analysis was performed for the central comparison between kick and wipe gestures. Comparisons involving loading condition, starting position, and demographic subgroups remained descriptive because the study was not designed or powered for formal subgroup inference. In addition, the number of analyzed participants (n=56) remained below the target of approximately 69 indicated by the sample-size estimation, because no valid recording remained for 13 of the recorded participants, mainly due to sensor-related problems. Since this estimation served only as a pragmatic recruitment target for a descriptive, reference-generating study, and the central comparison between kick and wipe gestures was additionally confirmed at the participant level with large and consistent effect sizes, the reduced sample is not expected to compromise the reported reference profiles.

Fifth, the demographic distribution was broad but not fully balanced. In particular, the 70+ age group included only two participants. The lower sagittal RoM observed in this subgroup should therefore not be generalized as an age effect. Future work should include a larger number of older participants to determine whether age-specific adaptations in movement amplitude, velocity, or postural control are relevant for contactless tailgate activation.

Finally, the present study focused on the biomechanical characterization of the human movement itself. Vehicle-integrated radar data were recorded during the experimental campaign, but the present manuscript does not include a synchronized comparison between IMU-derived kinematics and radar sensor responses. As a result, the relationship between joint-level movement execution, segment-level trajectories, and radar-specific observables such as Doppler signatures and range–Doppler patterns remains outside the scope of this study. Future work will link the synchronized radar recordings to the IMU-derived reference profiles to determine how specific kinematic features are reflected in vehicle-integrated radar signals, exploiting that radar-based systems observe the movement from a fixed vehicle-integrated perspective and are sensitive to the spatial relationship between the movement trajectory and the sensor-target direction [10,11]. Such multimodal datasets can further support the development of standardized test procedures, sensor simulation models, and future camera-based or markerless motion-capture approaches for exterior gesture recognition.

## 5. Conclusions

This study established wearable IMU-derived kinematic reference profiles of lower-limb kick and wipe gestures used for contactless automotive tailgate activation, based on N=6879 segmented movements from n=56 participants. Both gesture types shared a common sagittal baseline structure, while wipe gestures showed substantially larger hip abduction/adduction and ankle internal/external rotation. A participant-level confirmatory comparison supported this distinction, with significantly larger non-sagittal hip and ankle rotation for the wipe gesture in every participant (p<0.001). This identifies the wipe gesture as a distinct lower-limb HMI movement pattern rather than a lateral variant of the kick.

The reference profiles provide a wearable-sensor-derived kinematic basis for defining realistic human target trajectories, designing repeatable biomechanical test procedures and evaluating gesture-recognition sensor systems. Descriptive comparisons suggested that the main kinematic structure was broadly similar across loading conditions and most demographic subgroups. Because these comparisons were not formally tested, this observation should be confirmed in future studies with balanced subgroup sizes.

Future work will link the IMU-derived profiles to the synchronously recorded vehicle-integrated radar data, enabling direct analysis of how joint-level and segment-level movement features map onto radar-specific observables and supporting multimodal evaluation approaches for future exterior gesture-recognition systems.

## Figures and Tables

**Table 1:** Lower-body anthropometric characteristics of study participants. Values are reported as mean ± standard deviation.

Measurement [cm]	Male	Female
Hip width	29.5±3.3	27.8±3.4
Hip circumference	98.7±9.7	97.9±13.9
Upper leg length	42.2±4.5	37.4±4.6
Upper leg circumference	50.9±5.1	50.7±4.9
Knee circumference	39.1±2.9	37.9±3.0
Lower leg length	44.7±4.0	41.5±3.6
Lower leg circumference	39.4±2.7	36.8±2.6
Ankle circumference	28.5±3.3	27.6±3.1
Shoe length	31.3±1.2	27.4±1.8
Shoe width	11.8±0.5	10.6±0.6
Foot height	10.8±1.3	10.2±1.6

**Table 2:** Age and sex distribution of study participants (n=56).

Sex/Age	<30	30–39	40–49	50–59	60–69	70+
Male	7	4	8	16	5	2
Female	2	3	4	4	1	0
Total	9	7	12	20	6	2

**Table 3:** Data flow from recorded participants to the final movement dataset.

Processing Stage	Count
Recorded participants	69
Excluded, no valid recording (sensor-related problems)	13
Analyzed participants	56
Recordings excluded within analyzed participants	26
Final recorded and segmented movements	6879
Kick (unloaded/loaded)	1923/1600
Wipe (unloaded/loaded)	1778/1578
Total (unloaded/loaded)	3701/3178

**Table 4:** Loading matrices of the gesture-specific PCA for the first four principal components. Bold values indicate loadings with $|v_{jk}|\geq 0.30$, which were used as a qualitative criterion to identify dominant contributors and not as a statistical significance threshold. Abbreviations: F/E, flexion/extension; A/A, abduction/adduction; I/ER, internal/external rotation.

	Kick				Wipe			
**DoF**	**PC1**	**PC2**	**PC3**	**PC4**	**PC1**	**PC2**	**PC3**	**PC4**
Hip F/E	**0.52**	**−0.43**	−0.21	**0.62**	**−0.45**	**0.48**	−0.17	−0.23
Hip A/A	0.08	−0.03	**0.36**	0.19	−0.04	0.06	−0.19	**0.79**
Hip I/ER	0.09	−0.07	−0.05	0.07	−0.07	0.08	0.04	−0.27
Knee F/E	**0.80**	**0.52**	0.04	−0.27	**0.36**	**0.86**	0.03	0.05
Knee A/A	−0.02	0.01	−0.06	0.03	0.02	0.01	−0.01	−0.14
Knee I/ER	−0.06	0.05	0.05	−0.01	0.07	−0.03	0.19	−0.29
Ankle F/E	−0.24	**0.71**	0.03	**0.63**	**0.76**	−0.08	0.16	−0.01
Ankle A/A	−0.09	0.13	**−0.50**	**−0.30**	0.20	−0.09	**−0.39**	**−0.38**
Ankle I/ER	0.03	−0.09	**0.75**	−0.12	−0.18	0.06	**0.84**	0.03
Explained variance (%)	44.8	35.0	5.7	4.6	36.9	30.9	11.3	7.3

**Table 5:** Descriptive comparison of joint RoM between unloaded and loaded conditions. Relative differences are expressed with respect to the unloaded condition. Abbreviations: F/E, flexion/extension; A/A, abduction/adduction; I/ER, internal/external rotation.

DoF	Unloaded [°]	Loaded [°]	Abs. diff. [°]	Rel. diff. [%]
Hip F/E	26.3	26.1	−0.2	−0.8
Hip A/A	12.3	11.9	−0.4	−3.3
Hip I/ER	10.1	10.3	0.2	2.0
Knee F/E	44.1	44.3	0.2	0.5
Knee A/A	6.7	7.0	0.3	4.5
Knee I/ER	10.9	11.6	0.7	6.4
Ankle F/E	32.4	33.3	0.9	2.8
Ankle A/A	13.5	13.7	0.2	1.5
Ankle I/ER	12.2	12.6	0.4	3.3

**Table 6:** Descriptive mean RoM values of selected DoFs by sex and age group. Values are reported in degrees and were not subjected to inferential statistical testing. Abbreviations: K, kick; W, wipe; F/E, flexion/extension; A/A, abduction/adduction; I/ER, internal/external rotation.

	Hip F/E		Hip A/A		Hip I/ER		Knee F/E		Ankle F/E		Ankle I/ER	
**Group**	**K**	**W**	**K**	**W**	**K**	**W**	**K**	**W**	**K**	**W**	**K**	**W**
Male (n=42)	25.7	25.5	7.7	17.0	9.5	11.5	45.6	42.0	32.5	32.2	8.7	17.1
Female (n=14)	28.9	27.5	7.8	17.9	7.9	11.1	46.7	43.5	33.9	34.8	8.7	15.0
<30 (n=9)	29.7	28.4	6.9	18.7	9.7	12.5	46.6	43.4	32.5	33.5	7.3	15.7
30–39 (n=7)	27.2	26.2	7.1	16.5	10.0	12.4	46.8	43.2	35.8	35.0	9.1	17.4
40–49 (n=12)	24.6	24.3	8.1	15.6	8.7	10.4	43.9	41.0	34.2	34.0	9.7	16.2
50–59 (n=20)	26.1	25.8	8.0	17.3	9.0	11.4	47.5	42.4	31.5	32.3	8.6	17.4
60–69 (n=6)	25.7	25.0	6.9	18.0	8.6	10.7	44.4	42.8	31.2	29.4	8.2	16.4
70+ (n=2)	22.6	21.7	11.3	15.5	8.2	9.6	38.5	34.7	29.0	25.2	8.6	16.7

**Table 7:** Participant-level Wilcoxon signed-rank comparison of joint RoM between kick and wipe gestures across the n=56 participants. The kick and wipe columns report the per-participant mean RoM in degrees. Positive $r_{rb}$ indicates a larger RoM for wipe movements, negative $r_{rb}$ a larger RoM for kick movements. The *p*-values are Holm-corrected for the nine comparisons. Abbreviations: F/E, flexion/extension; A/A, abduction/adduction; I/ER, internal/external rotation.

DoF	Kick [°]	Wipe [°]	*z*	$p_{\mathrm{Holm}}$	$r_{\mathrm{rb}}$
Hip F/E	26.9	26.2	−1.79	0.148	−0.27
Hip A/A	8.0	17.8	−6.51	<0.001	1.00
Hip I/ER	9.2	11.5	−5.87	<0.001	0.90
Knee F/E	46.2	42.7	−3.64	<0.001	−0.56
Knee A/A	6.4	7.8	−5.86	<0.001	0.90
Knee I/ER	9.7	12.9	−6.39	<0.001	0.98
Ankle F/E	32.6	32.6	−0.05	0.961	−0.01
Ankle A/A	11.4	16.2	−6.51	<0.001	1.00
Ankle I/ER	8.8	17.1	−6.51	<0.001	1.00

## Data Availability

The datasets generated and analyzed in this study contain proprietary information of BMW Group and are not publicly available. Anonymized derived data supporting the findings of this study may be made available by the corresponding author upon reasonable request and subject to approval by BMW Group.

## Abbreviations

The following abbreviations are used in this manuscript:

A/A	Abduction/Adduction
DoF	Degree of Freedom
F/E	Flexion/Extension
HMI	Human–Machine Interaction
I/ER	Internal/External Rotation
IMU	Inertial Measurement Unit
IQR	Interquartile Range
PCA	Principal Component Analysis
PVE	Proportion of Variance Explained
RoM	Range of Motion
